# Circulating microRNAs from plasma as preclinical biomarkers of epileptogenesis and epilepsy

**DOI:** 10.1038/s41598-024-51357-4

**Published:** 2024-01-06

**Authors:** Kinga Szydlowska, Anna Bot, Karolina Nizinska, Maciej Olszewski, Katarzyna Lukasiuk

**Affiliations:** Laboratory of Epileptogenesis, Nencki Institute of Experimental Biology, Warsaw, Poland

**Keywords:** Neuroscience, Predictive markers

## Abstract

Epilepsy frequently develops as a result of brain insult; however, there are no tools allowing to predict which patients suffering from trauma will eventually develop epilepsy. microRNAs are interesting candidates for biomarkers, as several of them have been described to change their levels in the brains, and in the plasma of epileptic subjects. This study was conducted to evaluate the usefulness of plasma miRNAs as epileptogenesis/epilepsy biomarkers. In our studies, we used a rat model of temporal lobe epilepsy. An epileptogenic insult was status epilepticus evoked by stimulation of the left lateral nucleus of the amygdala. Next, animals were continuously video and EEG monitored for 3 months. Blood was collected at 14, 30, 60, and 90 days after stimulation. Blood plasma was separated and miRNA levels were analyzed. We compared miRNA levels between sham-operated and stimulated animals, and between animals with high and low numbers of seizures. We propose three miRNAs that could be biomarkers of epilepsy: miR-671, miR-9a-3p and miR-7a-5p. According to us, miR-206-5p is a potential biomarker of epileptogenesis, and miR-221-3p is a potential biomarker of epilepsy severity. We think that these five miRNAs can be considered in the future as potential treatment targets.

## Introduction

Epilepsy is one of the most common neurological disorders, affecting approximately 50 million people worldwide^1^. Temporal lobe epilepsy (TLE) is the most common focal intractable seizure disorder in adults^2^. Although electroencephalography (EEG) is the gold standard of epilepsy diagnostics, it is limited to special epilepsy monitoring units (UMUs), where continuous EEG recording is available^3^. Additionally, brain imaging is not a sufficient tool for epilepsy prognosis. This is why there is a great need for the identification of molecular epilepsy biomarkers that could become easy, affordable, and, most importantly, noninvasive diagnostic tools^3^. Of those, particularly interesting can be biomarkers found in body fluids^4^.

Molecular biomarkers are particularly attractive because they offer the possibility of rapid, simple, and inexpensive bedside tests where a set of one or more molecules are measured in a convenient biofluid, such as blood^3,5^. Various circulating molecules have been investigated as potential epilepsy biomarkers. Among them are S100β (S100 calcium binding protein B), HMGB1 (high mobility group box 1), neuron-specific enolase, ghrelin and des-acyl ghrelin and inflammatory cytokines^3,6–12^. However, many of them suffer from a lack of specificity for a single brain disorder or present technical challenges.

An emerging class of potential biomarkers for neurological diseases is microRNAs (miRNAs)^13^. miRNAs are short noncoding RNAs (20–24 bp) that function via sequence-specific binding to the 3’-untranslated regions (UTRs) of target mRNAs. MicroRNAs come from endogenous transcripts that form local hairpin structures, which are processed such that a single miRNA molecule accumulates from one arm of a hairpin precursor molecule^14^. It is estimated that a single miRNA can regulate hundreds of genes, which makes them one of the most powerful regulators of gene expression^15^. miRNAs are widely expressed in the brain, both in neuronal and glial cells^16,17^. This makes miRNAs great targets for the exploration of diagnostic and therapeutic strategies.

The last decade has brought multiple reports of miRNA dysregulation in the brains of epileptic patients and in different epilepsy animal models^18–24^. Several studies also prove that manipulating specific miRNAs can affect seizure thresholds in animals^25,26^.

The first animal study to examine the effects of seizures on miRNA levels in blood appeared in 2010^27^, and in 2015, the first clinical studies were published on blood-based miRNAs for the diagnosis of epilepsy^28,29^. Recent studies indicate that circulating miRNAs might also be used to help determine drug resistance in patients with TLE^23^.

Despite some information regarding miRNA changes in the plasma of animals in epilepsy models, we are still missing detailed information describing the long-term course of changes during epileptogenesis and epilepsy. This information may point to potential diagnostic biomarkers for epilepsy and targets for intervention during epileptogenesis. Therefore, we designed a complex study in which we measured the levels of miRNA in the plasma of rats at 14, 30, 60 and 90 days following the induction of epilepsy by status epilepticus (SE) evoked by electrical stimulation of the amygdala. We analyzed miRNA signatures in plasma depending only on time from epilepsy induction but also in relation to the stage of epilepsy development (presymptomatic vs symptomatic phase) and epilepsy phenotype in epileptic animals (low vs. high seizure number). We selected five miRNAs that could be potential biomarkers of epileptogenesis (miR-671, miR-9a-3p, miR-7a-5p), epilepsy (miR-206-5p), and epilepsy severity (miR-221-3p).

## Methods

### Animal surgery and status epilepticus induction

All animal procedures were approved by the Ist Local Ethical Committee on Animal Research in Warsaw (permit no. 483/2013, and 737/2015) and conducted in accordance with the guidelines established by The European Council Directives 2010/63/EU and ARRIVE guidelines. Adult male Sprague‒Dawley rats (270–320 g) were housed under controlled conditions (24 °C, humidity 50–60%, 12/12 h light–dark cycle) with food and water available ad libitum. Animals were housed in pairs. The environment was enriched by using various toys and snacks, which were changed every week. Experimentators spent extensive time with all the animals outside the experimental setting to decrease the stress of animals. Rats were subjected to regular handling every other day for 10 min. Additionally, to decrease the stress of rats in the experiment, they were first trained to being transported between specific rooms and used to handling during all the procedures.

The amygdala stimulation model of temporal lobe epilepsy was used in this study as previously described with some modifications^30^. Surgery was performed under isoflurane anesthesia (2–2.5% in 100% O_2_), followed by the injection of butorphanol (Butomidor, Richter Pharma AG, Wells, Austria; 0.5 mg/kg i.p.) for analgesia. A stimulating and recording bipolar wire electrode (Plastic One Inc., Roanoke, VA, # E363-3-2 WT-SPC) was implanted into the left lateral nucleus of the amygdala 3.6 mm posterior and 5.0 mm lateral to bregma and 6.5 mm ventral to the surface of the brain. A stainless steel screw electrode (Plastic One Inc., Roanoke, VA, #E363/20) was implanted contralaterally into the skull over the right frontal cortex (3.0 mm anterior and 2.0 mm lateral to bregma) as a surface EEG recording electrode. Two stainless steel screw electrodes were placed bilaterally over the cerebellum (10.0 mm posterior and 2.0 mm lateral to bregma) as grounding and reference electrodes. The contacts of all electrodes were placed in a multichannel electrode pedestal (Plastic One Inc., Roanoke, VA, #MS363), which was attached to the skull with dental acrylate (Duracryl Plus). After two weeks of recovery, animals were electrically stimulated via the intra-amygdala electrode to evoke SE. Stimulation consisted of a 100-ms train of 1-ms biphasic square-wave pulses (400 μA peak to peak) delivered at 60 Hz every 0.5 s for 30 min. If the animal did not enter SE, stimulation was continued for an additional 10 min. The SE was stopped 1 h after stimulation via an intraperitoneal injection of diazepam (20 mg/kg). If the first dose of diazepam did not suppress SE, the animal received subsequent doses of diazepam at 5 mg/kg. All stimulated animals were moved just after the stimulation to VIDEO-EEG monitoring units to confirm development of self-sustained status epilepticus and later to confirm the effect of i.p. diazepam injection to stop status epilepticus. Time-matched control animals had electrodes implanted but did not receive electrical stimulation. Due to extensive animal welfare improvements, the survival rate in our experimental model was very good and in all experimental cohorts one animal passed away during surgery and one after diazepam injection.

Rats were monitored with VIDEO-EEG (Comet EEG, Grass Technologies, West Warwick, RI; Panasonic WV-CP480) continuously from the moment of stimulation until the end of week 5 of the experiment (1st vEEG), from week 11 to week 16 (2nd vEEG) and starting at week 27 until the end of the experiment (3rd vEEG). Spontaneous seizures were identified from EEG recordings by browsing the EEG manually on the computer screen. An electrographic seizure was defined as a high-frequency (> 8 Hz), high-amplitude (> 2 × baseline) discharge lasting for at least 5 s. The latency to the first spontaneous seizure, number and frequency of seizures, and number of epileptic animals in each group were evaluated.

**Figure 1:**
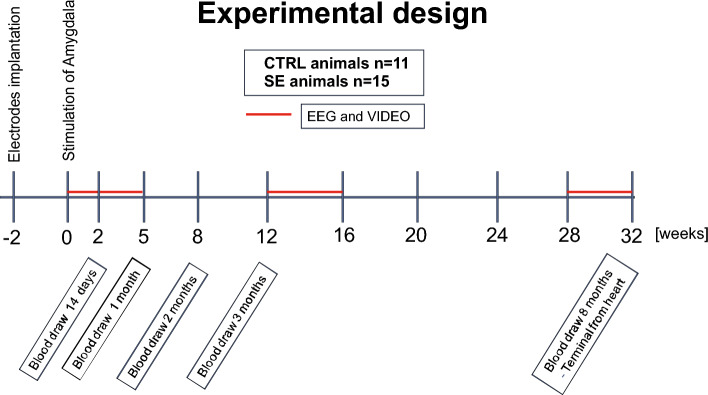
Experimental design. The whole experiment lasted for 224 days (8 months). Blood from the tail vein was drawn at 14 days, 30 days, 60 days and 90 days. VIDEO-EEG was monitored 3 times for 30 days during the whole course of the experiment.

The discovery cohort was prepared in two rounds. The first group of animals consisted of 6 control and 8 stimulated rats, and the second group consisted of 5 control and 7 stimulated animals. The validation cohort was prepared in four rounds, consisting of 2 control and 3 stimulated rats each. Experimental conditions were identical to those in the discovery cohorts of animals. In total, the discovery cohort consisted of 11 control and 15 stimulated animals, and the validation cohort consisted of 8 controls and 12 stimulated rats. The experimental design is presented in Fig. 1.

### Blood collection and plasma separation

Blood was drawn from restrained rats (#RSTR553N, Kent Scientific) at 14 days, 30 days, 60 days, and 90 days after SE. The blood was always drawn between 9 and 11 am. Every time the blood was drown interchangeably from control and stimulated group, and animals were chosen at a random order. Blood samples were collected from the tail vein in tubes coated with 1 mg ${K}_{2}$ EDTA (#363706, BD Vacutainer, USA). Within minutes after the blood draw, the blood was centrifuged at 1500 *rpm* for 5 min at + 4 °C. Plasma was later transferred to a new tube, aliquoted into 50 µl portions, snap frozen using dry ice, and stored at − 80 °C until use.

### miRNA isolation and profiling

miRNA was isolated from 50 µl of plasma using the miRCURY RNA isolation kit—Biofluids (#300112, Woburn, MA, Exiqon). The whole miRNA isolation procedure was performed as described in the producer’s manual (v. 1.7). Additionally, 1 µg of MS2 RNA (#10165948001, Mannheim, Germany, Roche Diagnostics GmbH) was used to increase miRNA yield during RNA isolation. Spike-in controls (miRCURY LNA Universal R microRNA PCR, RNA Spike-in Kit, #203203, Exiqon) were used as the internal microarray control. At the end of the procedure, RNA was eluted in 50 µl of RNAse-free water, aliquoted, and stored at − 80 °C.

miRNA levels were profiled using miRNA 4.1 Array Strip (#902404, Santa Clara, CA, Affymetrix). The procedure was performed according to the manufacturer’s recommendations. Eight microliters of RNA was used to perform a poly-A-tailing reaction (AF-902134 GA HWS Kit for miRNA Arrays, Affymetrix). Hybridization was performed for 20 h at + 48 °C (AF-900454 GeneChip Hybridization Control Kit, Affymetrix). It was followed by the wash and stain protocol (AF-901910 FlashTagTM Biotin HSR RNA Labeling Kit, Affymetrix). The whole procedure was performed using the GeneAtlas™ instrument from Affymetrix (#00-0393).

The data discussed in this publication have been deposited in NCBI's Gene Expression Omnibus^31^ and are accessible through GEO Series accession number GSE241756 (https://www.ncbi.nlm.nih.gov/geo/query/acc.cgi?acc=GSE241756).

### Real-time PCR

Alterations in the levels of selected miRNAs detected with microarrays were validated using the TaqMan system from Applied Biosystems by Life Technologies. In the discovery cohort, the same miRNA extracts were used for microarray experiments. cDNA synthesis was performed using a TaqMan MicroRNA Reverse Transcription Kit (#4366597, Vilnius, LT, Applied Biosystems by Life Technologies) and a nexus gradient Mastercycler (#6331, Eppendorf AG).

The PCR was performed using TaqMan Fast Universal PCR Master Mix (#4367846, Foster City, CA, Applied Biosystems by Life Technologies) and MicroAmp Fast Optical 96-Well reaction plates (#4346906, Applied Biosystems by Life Technologies) in a StepOnePlus System (Thermo Fisher Scientific). The primers used were as follows: miR-344a-5p: 4440886_463878_mat; miR-671: 4427975_002322; miR-3557-5p: 4440886_462825_mat; miR-1843b-5p: 4440886_476825_mat; miR-325-3p: 4427975_002510; miR-341: 4440886_461925_mat; miR-9a-3p = miR-9*: 4427975_002231; miR-3549: 4440886_465095_mat; miR-337-3p: 4440886_000193; miR-7a-5p: 4427975_000268; miR-218a-2-3p: 4427975_002294; miR-3593-5p: 4440886_462108_mat; miR-3556a: 4440886_463403_mat; miR-194-3p = miR-194*: 4427975_463169_mat; miR-206-5p = miR-206*: 4440886_463430_mat; miR-142-3p: 4427975_000464; miR-221-3p: 4427975_000524; miR-301a-3p: 4427975_000528. Data were analyzed using StepOne Software v2.3. The comparative Ct method (ΔΔCt method) was selected for analysis and performed according to ThermoFisher recommendations. As a reference, we used miRNA-301a-3p, which was selected as the most stable in our experiment, using the NormFinder algorithm^32^.

### Data analysis

Data analysis of the miRNA 4.1 Array Strips identifying up- and downregulated miRNAs was performed with RStudio (R v. 3.3.2) using Bioconductor^33^, oligo and limma packages. The microarrays were normalized with the Robust Multiarray Average (RMA) algorithm (oligo package version 1.22.0)^34^. For the expression analysis, the calculated p values were based on moderated t-statistics. Furthermore, the Benjamini and Hochberg multiple testing adjustment method was applied to the p values (FDR—false discovery rate). One-way ANOVA was used to establish miRNAs with differential levels between groups.

The correlation, cluster, and principal component analyses were performed in RStudio (R version 3.3.2)^35^. Principal component analysis (PCA) was performed on microarray probes and illustrated on PCA plots (splom) using the tidyverse and scatterplot3d packages. For the heatmap clustering of miRNAs with significantly different expression (p < 0.05) between all epileptic and sham-operated control animals, miRNAs were ordered with the clustering complete-linkage method and the Pearson correlation distance measure. The heatmap diagrams were generated with the gplots package. The Pearson correlation test was used to analyze the correlations between miRNAs with significantly different expression levels (p < 0.05) between stimulated and sham-operated control animals. The fuzzy c-means algorithm implemented in the Mfuzz package^36^ was used to perform clusterization on all probes.

### Statistics

The statistical analysis of Real-time PCR data was performed using Graph Pad Prism (v.8, GraphPad Software, USA). One-way ANOVA with the Bonferroni correction was used. The statistical significance was determined as follows: p < 0.05 “*”, p < 0.01, “**”, p < 0.001 “***”.

### Ethics approval and consent to participate

All animal procedures were approved by the Ist Local Ethical Committee on Animal Research in Warsaw (permit no. 483/2013, and 737/2015) and conducted in accordance with the guidelines established by The European Council Directives 2010/63/EU.

## Results

### Differences between sham and stimulated animals

We evaluated the expression of miRNA in the plasma of experimental animals consecutively at 14, 30, 60, and 90 days following amygdala stimulation-induced SE and time-matched sham controls. The experiment was carried out according to the experimental design presented in Fig. 1.

**Figure 2:**
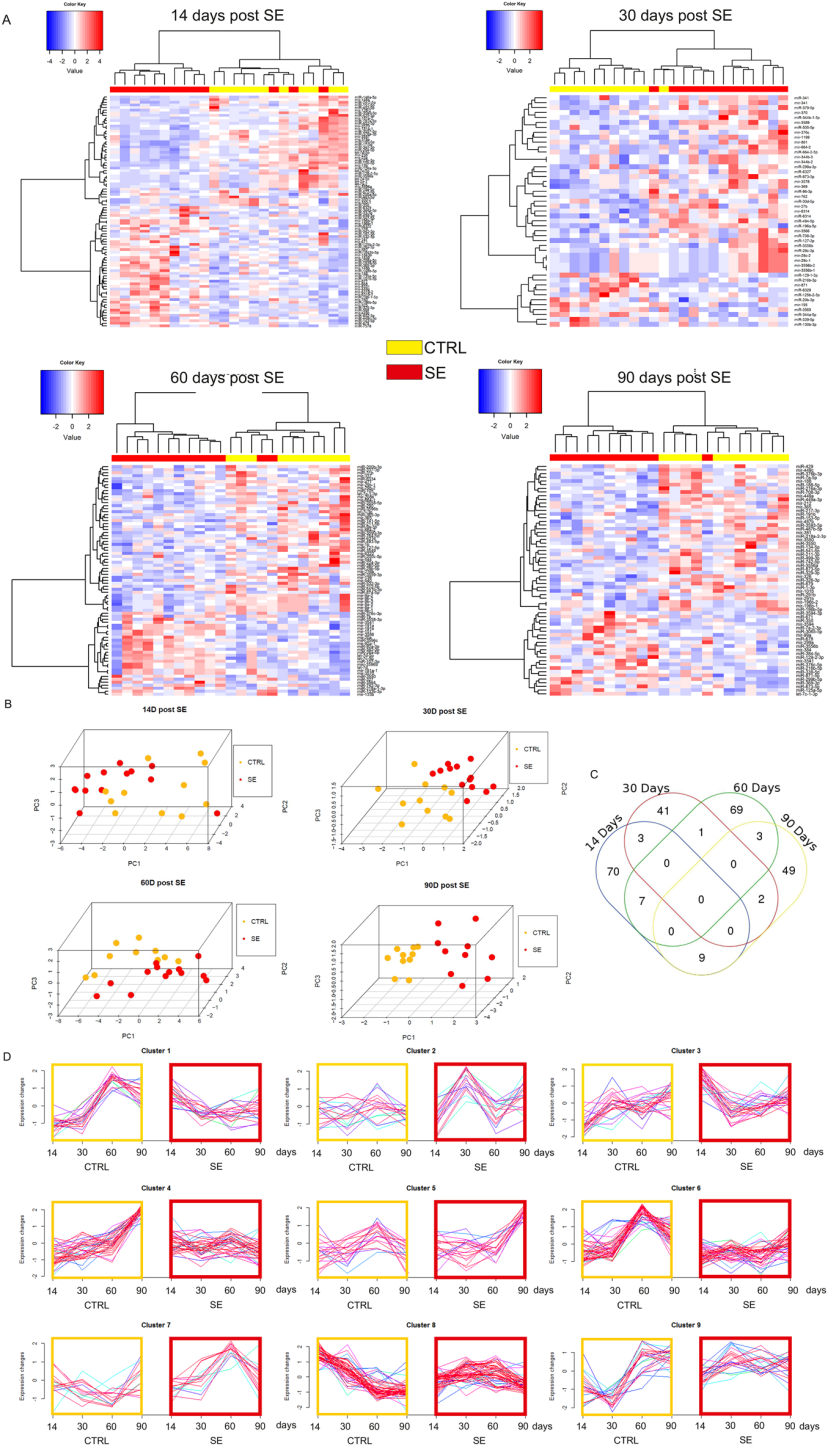
miRNA expression profiles in the plasma of epileptic and control animals at different times after SE. (**A**) Heatmaps present miRNAs with altered expression at 4 tested time points: 14, 30, 60 and 90 days post-SE. Each column represents individual animals, and each row represents an individual miRNA. We identified statistically significant changes in the expression levels of several miRNAs at the p < 0.05 cutoff: at 14 days, there were 90 miRNAs; at 30 days, there were 47 miRNAs; at 60 days, there were 81 miRNAs; and at 90 days, there were 63 miRNAs. The red bar over the heatmap indicates SE animals, and the yellow color bar indicates CTRL animals. Colors on the heatmaps represent increased (red) or decreased (blue) expression of a given miRNA. The heatmap diagrams were generated with the gplots package version 3.1.3 (R version 3.3.2)^35^. The fuzzy c-means algorithm implemented in the Mfuzz package version 2.32.0^36^ was used to perform clusterization on all probes. (**B**) Principal component analysis (PCA) graphs show spatial arrangements between CTRL (black) and SE (red) animals. Each mark represents an individual animal. Note that epileptic animals are separated from the controls. (**C**) Venn diagram presents an overlay of miRNA expression levels between the 4 tested time points. (**D**) Clusters represent groups of miRNAs displaying similar expression profiles over time induced by status epilepticus. The colors of lines within the clusters indicate the membership values of the expression profile to the current cluster. Red and violet are high membership values, and blue and green are low membership values.

We identified statistically significant changes in the levels of several miRNAs at the p < 0.05 cutoff. At 14 days, there were 90 miRNAs, at 30 days 47 miRNAs, at 60 days 81 miRNAs, and at 90 days 63 miRNAs with altered levels. Heatmaps (Fig. 2A) revealed different signatures between control and stimulated animals. Principal component analysis (PCA) separated control and stimulated animals at all tested time points (Fig. 2B).

We did not identify miRNAs that would change their levels at all tested time points, and only a few had altered levels at more than one time point (Fig. 2C). This indicates that most alterations in miRNA levels are transient.

To visualize alterations in miRNA levels over time, we performed cluster analysis using the Mfuzz algorithm (Fig. 2D). We identified several patterns of changes in ensembles of miRNAs over time, both in sham-operated and stimulated animals. Interestingly, we observed that some miRNA ensembles fluctuate in control animals (e.g., Fig. 2D—for example, clusters 1, 6, and 9). Patterns of miRNA levels over time are altered in stimulated animals (e.g., Fig. 2D—clusters 2, 5, and 7). This proves that miRNA levels change over time in the plasma of healthy animals and that epilepsy significantly affects their levels.

Since we identified a large number of miRNAs with altered levels, we concentrated on miRNAs with significantly changed levels in both experimental groups of the discovery cohort. We used a p < 0.05 cutoff, and in total, we discovered 13 miRNAs at all tested time points. We identified 5 miRNAs at 14 days post-SE. The upregulated genes were miR-344a-5p (logFC = 0.34, p = 0029, AUC = 0.6783), miR-671 (logFC = 0.28, p = 0.0008, AUC = 0.7902), miR-1843b (logFC = 0.39, p = 0036, AUC = 0.8182), and miR-325-3p (logFC = 0.24, p = 0007, AUC = 0.8042), and the downregulated gene was miR-3557-5p (logFC = − 0.52, p = 0.152, AUC = 0.7522). At 30 days post-SE, only one miRNA was changed. MiR-341 (logFC = 0.34, p = 0.0001, AUC = 0.9371) was upregulated. At 60 days, all selected miRNAs were downregulated: miR-9a-3p (logFC = -0.16, p = 0.0070, AUC = 0.8308), miR-3549 (logFC = − 0.61, p = 0.0002, AUC = 0.8615), and miR-337-3p (logFC = − 0.34, p = 0.0052, AUC = 0.6846). At the last tested time point (90 days post SE), 4 miRNAs were downregulated: miR-7a-5p (logFC = − 0.25, p = 0.0014, AUC = 0.8926), miR-218a-2-3p (logFC = − 0.21, p = 0.0098, AUC = 0.8264), miR-3593-5p (logFC = − 0.37, p = 0.0003, AUC = 0.9256) and miR-3556a (logFC = − 0.53, p = 0.0001, AUC = 0.9752). We performed ROC analysis to assess the sensitivity and specificity of each miRNA change. Values of AUC (area under the curve) close to 1 indicate that all selected miRNAs are potentially good biomarkers of epilepsy.

**Figure 3:**
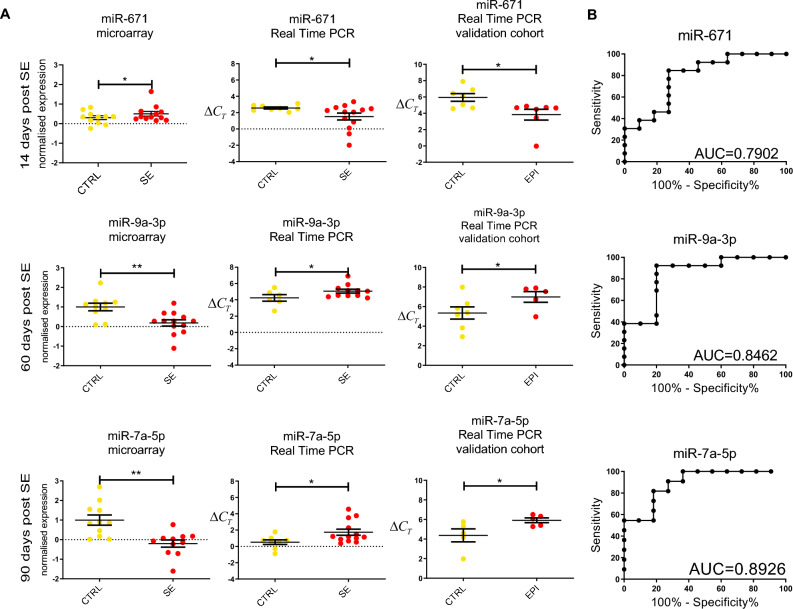
Selected biomarkers of epileptogenesis. (**A**) Detailed analysis of microarray results and their validation with real-time PCR allowed us to choose 3 potential biomarkers of epilepsy: miR-671 at 14 days post-SE, miR-9a-3p at 60 days post-SE and miR-7a-5p at 90 days post-SE. Microarray results are presented as the normalized expression (a higher value means higher expression). Real-time PCR results are presented as ΔCt values (values higher than the control mean decreased levels of expression). (**B**) ROC analysis proved very good sensitivity and specificity of all selected miRNAs.

To validate our results, we performed a series of real-time PCR experiments using the TaqMan system from Thermo Fisher Scientific (Fig. 3A) using plasma from both discovery and validation cohorts. Out of the 13 previously selected miRNAs, we found 3 miRNAs: miR-671 from 14 days post-SE, miR-9a-3p from 60 days post-SE, and miR-7a-5p from 90 days post-SE, which gave us similar, significant results in all 3 independent tests: microarrays, real-time PCR on the experimental cohort, and real-time PCR on the validation cohort. ROC analysis for these 3 miRNAs showed good sensitivity and specificity (AUC = 0.79 [miR-671], 0.85 [miR-9a-3p], and 0.89 [miR-7a-5p]) (Fig. 3B) Taking all these data into consideration, we propose that miR-671, miR-9a-3p and miR-7a-5p are potentially good biomarkers differentiating sham from stimulated animals.

### Differences between epileptic and nonepileptic stimulated animals

All stimulated animals developed epilepsy, diagnosed by the presence of spontaneous seizures at times following stimulation. However, the latency to the first spontaneous seizure differed between animals. This provided an opportunity to study differences in miRNA levels in epileptic vs. nonepileptic animals. At 14 days post SE, 7 animals had spontaneous seizures, whereas 6 animals were in the presymptomatic phase. At 30 days post-SE, 9 animals developed epilepsy, and 4 animals did not. At the 60- and 90-day time points, all stimulated animals were diagnosed with epilepsy. We analyzed differences in miRNA levels in sham animals vs. epileptic animals vs. presymptomatic animals.

**Figure 4:**
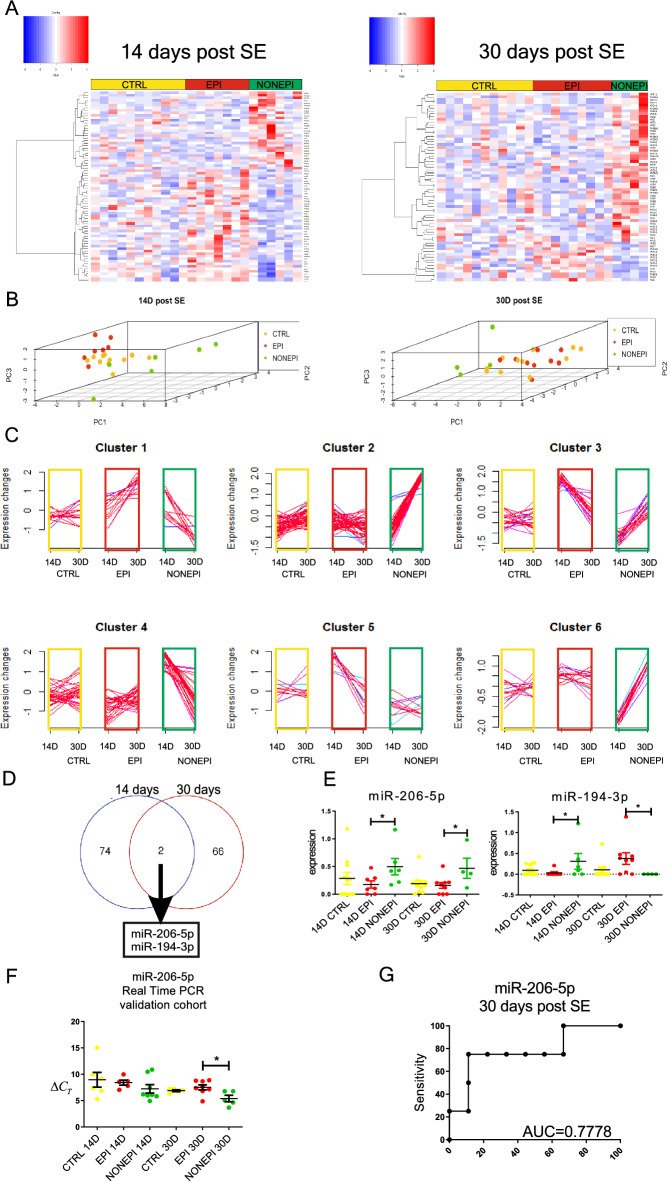
miRNA expression profiles in symptomatic and asymptomatic phases. Selection of epileptogenesis biomarkers. (**A**) Heatmaps present miRNAs with altered expression at 14 and 30 days post-SE, where we could select animals that did not suffer from seizures (NONEPI) and those that developed epilepsy (EPI). Each column represents individual animals, and each row represents an individual miRNA. We identified statistically significant changes in the expression levels of several miRNAs at the p < 0.05 cutoff. The red bar over the heatmap indicates EPI animals, the green bar indicates NONEPI animals, and the yellow color bar indicates CTRL animals. Colors on the heatmaps represent increased (red) or decreased (blue) expression of a given miRNA. The heatmap diagrams were generated with the gplots package version 3.1.3 (R version 3.3.2)^35^. The fuzzy c-means algorithm implemented in the Mfuzz package version 2.32.0^36^ was used to perform clusterization on all probes. (**B**) Principal component analysis (PCA) graphs show spatial arrangements between CTRL (black), NONEPI (green), and EPI (red) animals. Each mark represents an individual animal. Note that epileptic animals are separated from nonepileptic animals. (**C**) Clusters represent groups of miRNAs displaying similar expression profiles over time induced by epileptogenesis (green box) or epilepsy (red box). The colors of lines within the clusters indicate the membership values of the expression profile to the current cluster. Red and violet are high membership values, and blue and green are low membership values. (**D**) Venn diagram presents an overlay of miRNA expression levels between the 2 tested time points. (**E**) Based on the Venn diagram, we selected 2 miRNAs, miR-206-5p and miR-194-3p, that differ nonepileptic from epileptic animals. (**F**) We also validated our discovery in plasma samples from 4 independent validation cohorts of animals. We confirmed the distinctive change in miR-206-5p at 30 days post-SE in animals that did not develop seizures. Real-time PCR results are presented as ΔCt values (values higher than the control indicate a decreased level of expression). (**G**) ROC analysis confirmed that the sensitivity and specificity of miR-206-5p is good, and it can be considered a biomarker of epileptogenesis.

At 14 days post SE, 76 miRNAs differed between epileptic and presymptomatic animals (Fig. 4A), while at 30 days post SE, there was a difference in the levels of 68 miRNAs between epileptic and presymptomatic animals (Fig. 4A) (p < 0.05 cut off).

PCA clearly separated sham, epileptic and presymptomatic animals (Fig. 4B). Epileptic and presymptomatic animals were localized in opposite places in the 3D space in relation to sham animals. This suggests that the presymptomatic phase is not just a transitional phase before epilepsy diagnosis but also has specific characteristics.

We also compared the patterns of miRNAs differentiating between epileptic and presymptomatic animals over time in sham vs. epileptic vs. presymptomatic animals using the Mfuzz clustering algorithm (Fig. 4C). We observed that the levels of these miRNAs are relatively stable in sham animals, whereas in epileptic and presymptomatic animals, there are profound changes in levels over time. Interestingly, many of these changes occur in opposite directions in epileptic animals compared with presymptomatic animals. This is strong evidence of wide differences between the presymptomatic and symptomatic phases in epilepsy progression. It also indicates that in presymptomatic animals, there are several specific molecular processes being activated not only in the brain but also in the plasma of animals.

As a further step of our analysis, we selected miR-206-5p and miR-194-3p as miRNAs, whose levels changed similarly at both analyzed time points (Fig. 4D). Both miRNAs presented increased presymptomatic levels compared to epileptic animals at 14 days post-SE (Fig. 4E). At 30 days, miR-206-5p expression in presymptomatic animals was still elevated, whereas miR-194-3p was decreased. We later validated results obtained by microarrays using real-time PCR (TaqMan system). Since the detected level of miR-194-3p was extremely low, we thought it did not fulfill the requirement for being a good candidate biomarker. Thus, for further analysis, we focused solely on miR-206-5p. Real-time PCR for miR-206-5p confirmed our previous observation that its expression is increased at 30 days post-SE in presymptomatic animals (Fig. 4F). ROC analysis (Fig. 4G) assessed miR-206-5p sensitivity and specificity as very good, with AUC = 0.8. This indicates that miR-206-5p is a good candidate biomarker for preclinical studies differentiating between the symptomatic and presymptomatic phases of epilepsy.

### Differences between epileptic animals with low vs. high numbers of seizures

**Figure 5:**
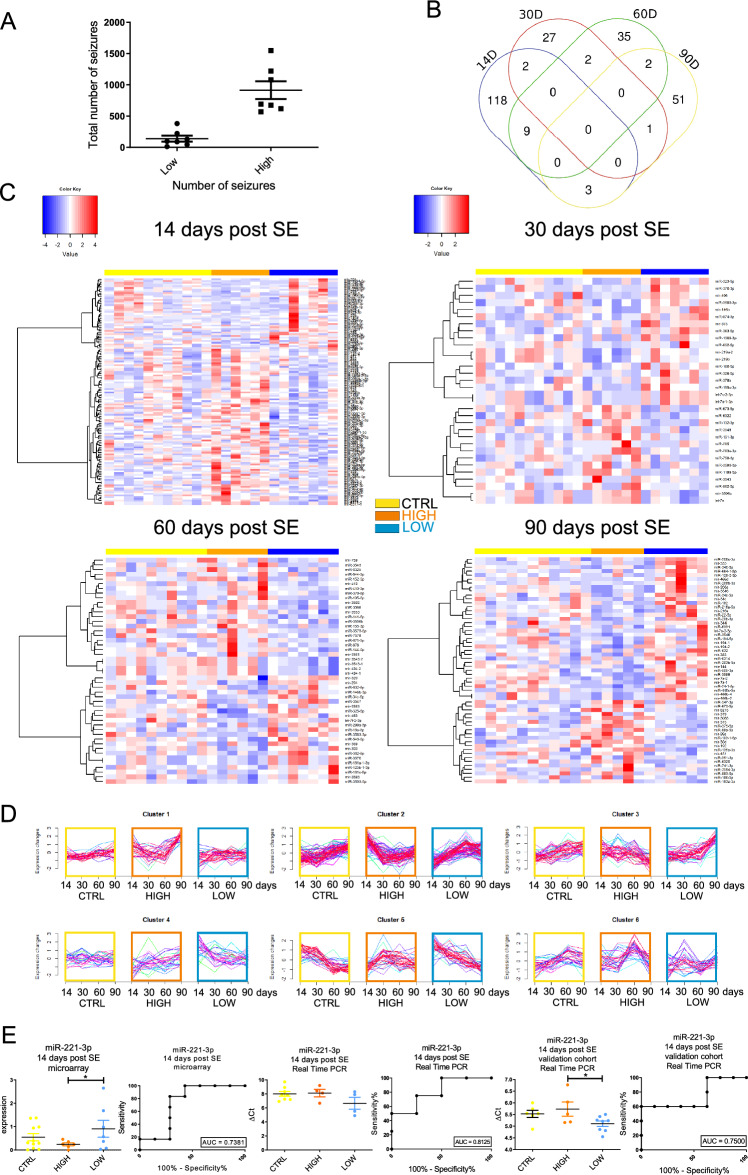
miRNA expression profiles in the plasma of animals with high and low numbers of seizures. (**A**) The number of seizures in stimulated animals varied greatly. Within the stimulated group, we can distinguish two subgroups with low (median = 95.0 + / − 47.58; LOW) and high (median = 692.8 + / − 141.4; HIGH) numbers of seizures. (**B**) Venn diagram presents an overlay of significantly changing miRNAs between the 4 tested time points. (**C**) Heatmaps present miRNAs with altered expression at 4 tested time points: 14, 30, 60, and 90 days post-SE. Each column represents individual animals, and each row represents an individual miRNA. We identified statistically significant changes in the expression levels of several miRNAs at a p < 0.05 cutoff. The orange bar over the heatmap indicates animals with a high number of seizures, the blue color marks animals with a low number of seizures, and the yellow color bar indicates CTRL animals. Colors on the heatmaps represent increased (red) or decreased (blue) expression of a given miRNA. The heatmap diagrams were generated with the gplots package version 3.1.3 (R version 3.3.2)^35^. The fuzzy c-means algorithm implemented in the Mfuzz package version 2.32.0^36^ was used to perform clusterization on all probes. (**D**) Clusters represent groups of miRNAs displaying similar expression profiles over time induced by status epilepticus. The colors of lines within the clusters indicate the membership values of the expression profile to the current cluster. Red and violet are high membership values, and blue and green are low membership values. Yellow boxes highlight control animals, orange boxes animals with a high number of seizures, and blue boxes animals with a low number of seizures. (**E**) Detailed analysis of microarray results and their validation with real-time PCR allowed us to choose a potential biomarker of epilepsy severity – miR-221-3p 14 days post-SE. Microarray results are presented as the normalized expression (a higher value means higher expression). Real-time PCR results are presented as ΔCt values (values higher than the control mean decreased levels of expression). ROC analysis proved very good sensitivity and specificity of all selected miRNAs.

Epileptic animals in our experiment had variable seizure numbers; therefore, we could divide epileptic animals into two groups according to the median number of seizures (median 473.9). As a result, we obtained a group of epileptic animals with low (median = 95.0) and high (median = 692.8) numbers of seizures during 8 months after SE (Fig. 5A). We found a number of miRNAs differentiating between animals with low vs. high seizure numbers at all time points: 132 miRNAs at 14 days, 34 miRNAs at 30 days, 48 miRNAs at 60 days and 57 at 90 days post-SE (p < 0.05 cut off). Heatmaps for each time point show different signatures for plasma samples from animals with low and high numbers of seizures (Fig. 5C).

Interestingly, an increase or decrease in the levels of given miRNAs was usually transient. Comparison of miRNAs significantly changing levels at different time points revealed no miRNAs that would have changed expression at all tested time points (Fig. 5B). We only observed a single miRNA changing its level at 2 time points.

Mfuzz clustering analysis revealed very interesting patterns between control animals and animals with high and low numbers of seizures (Fig. 5D). What is clearly visible in the expression pattern over time is that in significantly changing miRNAs, the difference between high and low animals is usually the opposite. For example, in cluster 2, the level of miRNAs decreased over time in animals with a high number of seizures, whereas it increased over time in animals with a low number of seizures (Fig. 5D).

We selected 3 miRNAs for more detailed analysis. We found that miR-221-3p is the one that best distinguishes between animals with high and low numbers of seizures (Fig. 5E). The difference in real-time PCR analysis from the discovery cohort (CTRL n = 11, and SE n = 15) was not significant; however, it showed a strong tendency toward the same changes as observed in microarrays. We ran PCR on the same RNA as the microarrays. Analysis by real-time PCR in a validation cohort with a higher number of animals revealed a significant change in miRNA-221-3p levels. Taken together, these results convinced us that miR-221-3p is a good biomarker that can distinguish early in epilepsy development between animals with high and low numbers of seizures.

## Discussion

In this study, we present a set of miRNAs whose signatures induced by status epilepticus in plasma change over long periods. We identified miRNAs that can serve as putative biomarkers of an asymptomatic phase of epilepsy development and miRNAs that allow assessment of epilepsy severity by differentiating between animals with low vs. high seizure numbers. All the identified miRNAs can be potentially useful for preclinical studies.

Molecular biomarkers, such as miRNAs, which could be reliably detected in easily obtainable biofluids, could mean a breakthrough in epilepsy diagnosis^8,19,21,23,28,29,37–40^. Plasma is a good choice for diagnostic biofluids. It is easily obtainable, contrary to cerebrospinal fluid, and allows for avoiding clotting events, which can occur in serum and can create strong variability in miRNA profiles^41^.

Our study involved a dual-platform approach to discover TLE biomarkers of different phases of epilepsy development. The discovery phase of our experiments led to the identification of several miRNAs that changed levels between sham animals and animals at different stages of epilepsy development with different epilepsy phenotypes. These results were validated using TaqMan Real-time PCR as an alternative method using an additional animal cohort. This approach allowed us to propose three miRNAs whose levels change significantly after status epilepticus (miR-671, miR-9a-3p, miR-7a-5p), one as a biomarker of the presymptomatic phase (miR-206-5p) and one as a biomarker of epilepsy severity (miR-221-3p). This is the first such detailed, long-term study of circulating miRNA expression profiles in animal models of TLE.

To date, only a few papers have been published presenting changes in miRNA levels in blood or plasma after seizures and/or epilepsy induction. They present changes in miRNA levels 24 h after kainate injection^27^ or lithium and pilocarpine injection^42^. They identified several miRNAs with changed levels both in the brain and the blood. This led to the idea that there may be a “leakage” of miRNAs to the blood from the brain after seizures. Interestingly, similar miRNA changes were observed after seizures and after ischemia and intracerebral hemorrhage. This may indicate that these miRNAs may be markers of brain injury of different etiologies^27^.

Gorter et al. published the results of their study in which they checked the expression of miRNAs in the hippocampi of animals after SE induced by electrical stimulation of the hippocampus^43^. They performed a timeline analysis of changes during epileptogenesis and after epilepsy development at 1, 7, and 30 days post-SE. They selected 3 upregulated miRNAs, miR-146a-5p, miR-21-5p, and miR-142-5p, and their pattern of expression in plasma was similar to that observed in the brain. This study confirmed that the same miRNAs can be deregulated in the brain as in biofluids and can serve as biomarkers of epileptogenesis and epilepsy.

Roncon et al. examined miRNA expression profiles in the plasma of rats in the lithium-pilocarpine model at early and late latency phases and in a chronic phase of epilepsy using microarrays and discovered an increase in miR-9a-3p levels as a potential early epileptogenesis biomarker^20^.

Raoof et al. performed RNA sequencing from plasma samples of TLE patients, which included psychogenic nonepileptic seizures (PNES), 24 h after they experienced a seizure^44^. After profiling and validation, they identified miR-27a-3p, miR-328-3p, and miR-654-3p as potential symptomatic epilepsy biomarkers. The same miRNAs were detected in the plasma of animals that underwent intraamygdala injection of kainic acid.

Brennan et al. performed a study of miRNA profiling in plasma in three different animal models: kainate, pilocarpine, and perforant pathway stimulation^45^. The authors identified a set of 5 miRNAs that changed similarly in all tested models: miR-93-5p, miR-142-5p, miR-182-5p, miR-199a-3p and miR-574-3p. Validation studies found that miR-93-5p, miR-199a-3p, and miR-574-3p were also dysregulated in plasma from patients with intractable temporal lobe epilepsy, proving that similar changes in miRNA levels detected in animal models can be detected in human samples.

We identified three miRNAs regulated in plasma by SE: miR-671 upregulated at 14 days post-SE, miR-9a-3p downregulated at 60 days post-SE, and miR-7a-5p downregulated at 90 days post-SE. MiR-671 was previously found to be upregulated in the plasma of patients suffering from traumatic brain injury, which can induce epilepsy in animals and human patients^46,47^. Interestingly, miR-671 indirectly regulates miR-7 activity by targeting and reducing circular RNA ciRS-7 levels in HeLa cells^48^.

An increase in miR-9a-3p levels in the plasma of pilocarpine-induced epileptic rats was previously proposed as a biomarker of the early stages of epileptogenesis^20^. Roncon et al. showed an increase in miR-9a-3p levels only in the early phases of epileptogenesis (4 days post-SE), and at 8 days post-SE, it had already dropped, whereas in our study, the first time point tested was 14 days after SE, where we also did not detect a significant difference in miR-9a-3p levels^20^. In our model of electric amygdala stimulation, miR-9a-3p expression was downregulated at the chronic phase of epilepsy at 60 days post-SE. Roncon et al. did not detect an increase in miR-9a-3p at 60 days post-SE, as we did, and they did not check later time points. It is possible that the timeline of miR-9a-3p level increase is different in various animal epilepsy models and requires more detailed testing to fully understand how miR-9a-3p levels change over time. Nevertheless, this miRNA is of interest in future studies of epileptogenesis/epilepsy biomarkers. Interestingly, increased miR-9a-3p levels in human plasma samples were implicated in Alzheimer’s disease, frontotemporal dementia and Parkinson’s disease^49^. This may suggest that miR-9a-3p (miR-9*) can be considered a marker of general neurological damage. miR-7a-5p has been shown to be upregulated in extracellular vesicles from the brain in an animal model of traumatic brain injury^50^. In our previous study, we found that its level is decreased in the dentate gyrus of epileptic rats^19^ at the same time point. It can be concluded that all 3 miRNAs have potential as brain damage biomarkers.

In the next step, we focused on differentiating animals in presymptomatic and symptomatic phases of epilepsy. We identified 2 miRNAs, miR-206-5p and miR-194-3p, which were elevated in nonepileptic animals both at 14 and 30 days post-SE when compared to epileptic animals. Due to a low level of miR-194-3p expression, we decided that miR-194-3p is not a suitable candidate. Another candidate, miR-206, plays a role in cardiomyocyte function by regulating connexin 43 (Jin et al. 2018). miR-206 also showed increased levels in the serum of mild cognitive impairment (MCI) patients^51^. miR-206-3p expression was significantly upregulated in the hippocampus and cortex of APP/PS1 mice in the pathology of Alzheimer’s disease and influenced the expression of BDNF (brain-derived neurotrophic actor)^52^. We propose that miR-206-5p is a potentially good candidate biomarker of the asymptomatic phase of epilepsy since its levels rise in the plasma of stimulated animals that have not yet developed epilepsy.

We would like to stress that when analyzing miRNA level changes in plasma after SE, it is clear that epileptogenesis (asymptomatic phase) is a process that can be clearly distinguished from the symptomatic phase of epilepsy. This finding is in agreement with the results we obtained when analyzing brain miRNA levels in the dentate gyrus after SE induction^19^. Similar observations were evident in studies of circulating RNA levels in the plasma of pilocarpine-injected animals^20^ and in the plasma of animals after SE induced by electrical stimulation of the hippocampus^43^. This observation is direct proof that epileptogenesis can be identified by ongoing miRNA level changes and that they are good candidates for epileptogenesis biomarkers.

We found that the level of miR-221-3p is higher in animals with low seizure numbers, and therefore, it is a potential biomarker of epilepsy severity. Bencurova et al. showed that the level of miR221-3p was elevated in the rat hippocampus at the chronic stage in the pilocarpine model of epilepsy^38^. In agreement with our observations from plasma, miR-221-3p decreases in the cerebrospinal fluid and urine of children suffering from traumatic brain injury^53^ and is upregulated in the cerebrospinal fluid of ischemic stroke patients^54^. This means that miR-221-3p levels may depend on etiology and/or disease prognosis.

We performed biomarker selection using multiple animal groups and dual-platform selection. We think that the proper procedure of epilepsy biomarker identification should involve such a multilayered approach to allow for the selection of only those biomarkers that have the highest rate of change, are easy to detect and are reliably detected despite many differences that can be present in subsequent cohorts of animals. This observation is in agreement with human studies where miRNA profiling is performed. According to Raoof et al., using dual-platform and possibly dual-center approaches in miRNA profiling studies is crucial^44^. Only then will we be able to identify epilepsy biomarkers and potential targets for therapeutic intervention. Using single cohort experiments or pooled samples does not have strong diagnostic potential. Therefore, our experiments have an advantage over previously published studies searching for miRNAs in plasma in various epilepsy models as epilepsy biomarkers. Our study consisted of several experimental and validation animal groups. Additionally, the length of our study allowed us to examine various stages of asymptomatic and symptomatic phases of epilepsy in the animal model of temporal lobe epilepsy.

## Conclusions

Our complex, long-term study with multiple discovery and validation animal groups, using two platforms to test the expression of miRNA, allowed the discovery of SE-induced, presymptomatic, and epilepsy severity-related biomarkers. Although the mechanistic role of these miRNAs is not determined, they can be very useful as biomarkers of disease stage or severity. Most importantly, they can be valuable tools in preclinical studies and allow assessment of the disease's development or progression.

## Data Availability

The data discussed in this publication have been deposited in NCBI's Gene Expression Omnibus^31^ and are accessible through GEO Series accession number GSE241756 (https://www.ncbi.nlm.nih.gov/geo/query/acc.cgi?acc=GSE241756).

## References

[1] Banerjee PN, H. W. *Incidence and prevalence*. 2nd edn, (Wolters Kluwer Lippincott Williams & Wilkins, UK, 2008).

[2] Moshé SL, Perucca E, Ryvlin P, Tomson T (2015). Epilepsy: New advances. Lancet.

[3] Engel J (2013). Epilepsy biomarkers. Epilepsia.

[4] Hegde M, Lowenstein DH (2014). The search for circulating epilepsy biomarkers. Biomark. Med..

[5] Pitkänen A (2016). Advances in the development of biomarkers for epilepsy. Lancet Neurol..

[6] Walker LE (2017). Molecular isoforms of high-mobility group box 1 are mechanistic biomarkers for epilepsy. J. Clin. Invest..

[7] Lukasiuk K, Becker AJ (2014). Molecular biomarkers of epileptogenesis. Neurotherapeutics.

[8] Walker LE (2016). WONOEP appraisal: Molecular and cellular biomarkers for epilepsy. Epilepsia.

[9] Venereau E (2016). HMGB1 as biomarker and drug target. Pharmacol. Res..

[10] Marchiò M (2019). Decreased ghrelin and des-acyl ghrelin plasma levels in patients affected by pharmacoresistant epilepsy and maintained on the ketogenic diet. Clin. Nutr..

[11] Marchiò M (2018). High plasma levels of ghrelin and des-acyl ghrelin in responders to antiepileptic drugs. Neurology.

[12] Costa AM (2022). Prospective evaluation of ghrelin and des-acyl ghrelin plasma levels in children with newly diagnosed epilepsy: Evidence for reduced ghrelin-to-des-acyl ghrelin ratio in generalized epilepsies. J. Pers. Med..

[13] Rao P, Benito E, Fischer A (2013). MicroRNAs as biomarkers for CNS disease. Front. Mol. Neurosci..

[14] Ambros V (2003). A uniform system for microRNA annotation. RNA.

[15] Ebert MS, Sharp PA (2012). Roles for microRNAs in conferring robustness to biological processes. Cell.

[16] Jovičić A (2013). Comprehensive expression analyses of neural cell-type-specific miRNAs identify new determinants of the specification and maintenance of neuronal phenotypes. J. Neurosci..

[17] Sempere LF (2004). Expression profiling of mammalian microRNAs uncovers a subset of brain-expressed microRNAs with possible roles in murine and human neuronal differentiation. Genome Biol..

[18] McKiernan RC (2012). Reduced mature microRNA levels in association with dicer loss in human temporal lobe epilepsy with hippocampal sclerosis. PLoS One.

[19] Bot AM, Dębski KJ, Lukasiuk K (2013). Alterations in miRNA levels in the dentate gyrus in epileptic rats. PLoS One.

[20] Roncon P (2015). MicroRNA profiles in hippocampal granule cells and plasma of rats with pilocarpine-induced epilepsy–comparison with human epileptic samples. Sci. Rep..

[21] Raoof R (2017). Cerebrospinal fluid microRNAs are potential biomarkers of temporal lobe epilepsy and status epilepticus. Sci. Rep..

[22] Bencurova P (2017). MicroRNA and mesial temporal lobe epilepsy with hippocampal sclerosis: Whole miRNome profiling of human hippocampus. Epilepsia.

[23] De Benedittis S (2021). Circulating microRNA: The potential novel diagnostic biomarkers to predict drug resistance in temporal lobe epilepsy, a pilot study. Int. J. Mol. Sci..

[24] Mooney C, Becker BA, Raoof R, Henshall DC (2016). EpimiRBase: A comprehensive database of microRNA-epilepsy associations. Bioinformatics.

[25] Jimenez-Mateos EM (2012). Silencing microRNA-134 produces neuroprotective and prolonged seizure-suppressive effects. Nat. Med..

[26] Jimenez-Mateos EM (2011). miRNA Expression profile after status epilepticus and hippocampal neuroprotection by targeting miR-132. Am. J. Pathol..

[27] Liu DZ (2010). Brain and blood microRNA expression profiling of ischemic stroke, intracerebral hemorrhage, and kainate seizures. J. Cereb. Blood Flow Metab..

[28] Wang, J. *et al.* Circulating microRNAs are promising novel biomarkers for drug-resistant epilepsy. *Sci. Rep.* **5**(1). 10.1038/srep10201 (2015). 10.1038/srep10201 PMC4435024 25984652

[29] Wang J (2015). Genome-wide circulating microRNA expression profiling indicates biomarkers for epilepsy. Sci. Rep..

[30] Nizinska K (2021). Behavioral characteristics as potential biomarkers of the development and phenotype of epilepsy in a rat model of temporal lobe epilepsy. Sci. Rep..

[31] Edgar R, Domrachev M, Lash AE (2002). Gene expression omnibus: NCBI gene expression and hybridization array data repository. Nucleic Acids Res..

[32] Andersen CL, Jensen JL, Ørntoft TF (2004). Normalization of real-time quantitative reverse transcription-PCR data: A model-based variance estimation approach to identify genes suited for normalization, applied to bladder and colon cancer data sets. Cancer Res..

[33] Gentleman RC (2004). Bioconductor: Open software development for computational biology and bioinformatics. Genome Biol..

[34] Carvalho BS, Irizarry RA (2010). A framework for oligonucleotide microarray preprocessing. Bioinformatics.

[35] R_Core_Team. Vol. http://www.R-project.org/ (R Foundation for Statistical Computing, Vienna, 2012).

[36] Futschik & M. (2012).

[37] Henshall DC (2016). MicroRNAs in epilepsy: pathophysiology and clinical utility. Lancet Neurol..

[38] Bencurova P (2021). Dynamic miRNA changes during the process of epileptogenesis in an infantile and adult-onset model. Sci. Rep..

[39] Surges R (2016). Changes in serum miRNAs following generalized convulsive seizures in human mesial temporal lobe epilepsy. Biochem. Biophys. Res. Commun..

[40] Abou-Zeid A, Saad M, Soliman E (2011). MicroRNA 146a expression in rheumatoid arthritis: Association with tumor necrosis factor-alpha and disease activity. Genet Test. Mol. Biomarkers.

[41] van Vliet EA (2017). Standardization procedure for plasma biomarker analysis in rat models of epileptogenesis: Focus on circulating microRNAs. Epilepsia.

[42] Hu K (2011). Expression profile of microRNAs in rat hippocampus following lithium-pilocarpine-induced status epilepticus. Neurosci. Lett..

[43] Gorter JA (2014). Hippocampal subregion-specific microRNA expression during epileptogenesis in experimental temporal lobe epilepsy. Neurobiol. Dis..

[44] Brennan, G. P. *et al.* Genome-wide microRNA profiling of plasma from three different animal models identifies biomarkers of temporal lobe epilepsy. *Neurobiol. Dis.* **144**, 105048. 10.1016/j.celrep.2016.02.042 (2020). 10.1016/j.nbd.2020.105048 32800995

[45] Brennan GP (2016). Dual and opposing roles of MicroRNA-124 in epilepsy are mediated through inflammatory and NRSF-dependent gene networks. Cell Rep..

[46] Di Pietro V (2017). MicroRNAs as novel biomarkers for the diagnosis and prognosis of mild and severe traumatic brain injury. J. Neurotrauma.

[47] Pasinetti GM, Ho L, Dooley C, Abbi B, Lange G (2012). Select non-coding RNA in blood components provide novel clinically accessible biological surrogates for improved identification of traumatic brain injury in OEF/OIF Veterans. Am. J. Neurodegener Dis..

[48] Hansen TB (2013). Natural RNA circles function as efficient microRNA sponges. Nature.

[49] Sheinerman KS (2017). Circulating brain-enriched microRNAs as novel biomarkers for detection and differentiation of neurodegenerative diseases. Alzheimers Res. Ther..

[50] Harrison EB (2016). Traumatic brain injury increases levels of miR-21 in extracellular vesicles: Implications for neuroinflammation. FEBS Open Bio.

[51] Piscopo P (2018). MicroRNAs and mild cognitive impairment: A systematic review. Ageing Res. Rev..

[52] Wang CN (2017). The anti-dementia effects of donepezil involve miR-206-3p in the hippocampus and cortex. Biol. Pharm. Bull..

[53] Hicks SD (2018). Overlapping MicroRNA expression in saliva and cerebrospinal fluid accurately identifies pediatric traumatic brain injury. J. Neurotrauma.

[54] Sørensen SS, Nygaard AB, Nielsen MY, Jensen K, Christensen T (2014). miRNA expression profiles in cerebrospinal fluid and blood of patients with acute ischemic stroke. Transl. Stroke Res..

